# Exposure Profile to Traffic Related Pollution in Pediatric Age: A Biomonitoring Study

**DOI:** 10.3390/ijerph181910118

**Published:** 2021-09-26

**Authors:** Arianna Antonucci, Carmela Protano, Maria Luisa Astolfi, Vincenzo Mattei, Francesca Santilli, Stefano Martellucci, Matteo Vitali

**Affiliations:** 1 Department of Public Health and Infectious Diseases, “Sapienza” University of Rome, 00185 Rome, Italy; arianna.antonucci@uniroma1.it (A.A.); matteo.vitali@uniroma1.it (M.V.); 2 Department of Chemistry, “Sapienza” University of Rome, 00185 Rome, Italy; marialuisa.astolfi@uniroma1.it; 3 Biomedicine and Advanced Technologies Rieti Center, Sabina Universitas, 02100 Rieti, Italy; v.mattei@sabinauniversitas.it (V.M.); f.santilli@sabinauniversitas.it (F.S.); s.martellucci@sabinauniversitas.it (S.M.); 4 Department of Experimental Medicine, “Sapienza” University of Rome, 00185 Rome, Italy

**Keywords:** pediatric age, traffic related pollution, human biomonitoring, methyl tert-butyl ether, ethyl tert-butyl ether, tert-amyl methyl ether, diisopropyl ether, urinary biomarkers

## Abstract

The aim of this study was to trace an exposure profile to traffic-derived pollution during pediatric age. For this purpose, two biomonitoring campaigns for the determination of urinary (u-) methyl tert-butyl ether (MTBE), ethyl tert-butyl ether (ETBE), tert-amyl methyl ether (TAME), and diisopropyl ether (DIPE) were carried out in two different periods of the year (summer 2017 and winter 2018), among a large sample of healthy children (*n* = 736; 5–11 years old) living in rural and urban areas in central Italy. The quantification of u-MTBE, u-ETBE, u-TAME, and u-DIPE was performed by HS-SPME-GC/MS technique and information on participants was collected by a questionnaire. u-DIPE concentrations resulted always under the LOQ. u-TAME mean levels were similar in both seasons (18.7 ng L^−1^ in summer vs. 18.9 ng L^−1^ in winter), while u-MTBE and u-ETBE levels were, respectively, 69.9 and 423.5 ng L^−1^ (summer) and 53.3 and 66.2 ng L^−1^ (winter). Main predictors of urinary excretion resulted the time spent in motor vehicles, being male and younger.

## 1. Introduction

Based on emissions benefits, vehicle performance and existing regulations, ethers are the most used additives in gasoline blend [1,2]. Among them, methyl tert-butyl ether (MTBE), tert-amyl methyl ether (TAME), ethyl tert-butyl ether (ETBE), and di-isopropyl ether (DIPE) are those most frequently used in fuel production, to enhance the combustion process and reduce harmful exhaust emissions from motor vehicles [3,4]. The commercial production of MTBE started in 1973 in Europe and in 1979 in the United States. Italy was the first European country to add MTBE into gasoline, followed by other European countries in the mid-1980s [5]. The European Directive 98/70/EC [6] regulates the fuel quality for ensuring commercial gasoline harmonization across the EU. Over the years, in view of complying the EU integrated climate and energy policy, the content of MTBE in fuel was gradually increased up to 15%, aiming to reduce atmospheric levels of carbon monoxide and ozone. In 2003, the EU began to focus on renewable energy in transport fuels [7], shifting the ether production from MTBE to bio-ETBE, which is easier to blend than bio-ethanol. The subsequent Fuel Quality Directive [8] enhanced the permitted levels of ethers from 15% to 22% (*v/v*) and introduced mandatory requirements for the Member States to increase the use of biofuel to reduce greenhouse gas emissions by 6% by 2020. The actual ethers content of a specific fuel depends on petrol grade, oil company, and country. In Italy, the European Directive 2009/30/EC was implemented by the Legislative Decree 31 March 2011, which made mandatory the compliance with sustainability criteria for biofuels since January 2012 [9].

Although these oxygenated additives have been introduced to reduce the impact of vehicular emission on air quality, the massive use of MTBE in the past has led to a diffuse air pollution by this compound. Thus, many studies have been carried out in different countries to assess MTBE air levels; for example, the annual mean concentration of MTBE recorded in Helsinki (Finland) ranged from 1.1 to 2.8 μg m^−3^ [10], in Boston (USA) from 0.1 to 1 μg m^−3^ [11], in Zurich (Switzerland) from 0.4 to 0.7 μg m^−3^ [12], while in Milan (Italy) an annual median level of 0.68 μg m^−3^ was measured [13]. Besides this, Vainiotalo et al. found MTBE mean air concentrations in two Finnish self-service stations ranging from 70 to 1347 μg m^−3^ [14,15]. The levels differ widely depending on many factors, such as the period of the year, amount of gasolines sold, wind speed, exhaust emissions from passing traffic, and deliveries of gasoline to the station. With regard to ETBE, TAME, and DIPE, currently used in many fuels together with or instead of MTBE, few data are available and reported only for occupational settings. For example, Eitaki et al. [16] reported personal exposure levels of workers and airborne concentrations of ETBE at four gas stations respectively ranging from 0.01 to 0.28 ppm and from less than 0.01 to 4.12 ppm. Besides this, Campo et al. reported personal exposure levels of ETBE in petrol station workers and control subjects respectively equal to 3.1 and <0.8 µg m^−1^ (median values) [17]. Further, Vainiotalo et al. reported a geometric mean concentration of TAME equal to 0.98 mg m^−1^ as personal exposure level of a sample of Finnish tank lorry drivers [18].

The huge use of these compounds and the large quantities released into the environment has created widespread concern about exposure, both for the general population and in occupational settings, due to their toxicity. Indeed, the International Agency for Research on Cancer (IARC) classified MTBE as a possible cancer-causing agent [19], and asthmatic children, the elderly, and those with immunodeficiency are known to be at increased risk for toxicity [20]. Moreover, TAME, DIPE, and ETBE were found to be potential carcinogenic agents for various organs and tissues [21,22,23]. Exposure assessment has been performed by both air and biological monitoring. Indeed, a linear correlation was demonstrated between ethers’ air concentrations and their urinary (u-) levels, allowing their use as reliable biomarkers for assessing exposure to fuels and fuel exhausts [24,25]. Most studies were focused on the monitoring of u-MTBE, ETBE, TAME, and DIPE levels in exposed workers (urban policemen, service station workers, petrol tank lorry drivers, etc.) [18,24,26,27,28,29,30], while very few data are available on the exposure of subjects not professionally exposed [31,32,33], requiring further investigation. In this context, a high-risk group of population is represented by children [34], who are more susceptible than adults to airborne pollutants as demonstrated by some studies indicating that the prevalence of asthma, the most frequent chronic disease in children, is directly related to traffic pollution levels [35,36,37,38,39]. Thus, children exposure to airborne pollutants should be carefully assessed by targeted research.

The aim of this study was to trace an exposure profile to traffic-derived pollution during pediatric age. For this purpose, two biomonitoring campaigns for the determination of u-MTBE, u-ETBE, u-TAME, and u-DIPE were carried out in two different periods of the year (summer 2017 and winter 2018), among a sample of school-aged children attending six primary schools located in central Italy.

## 2. Materials and Methods

### 2.1. Study Population and Sampling Design

Two different sites in central Italy were individuated for the study: a rural area, at low population and traffic density, and an urban area, at medium population and traffic density [40].

A large sample of schoolchildren (*n* = 736; 5–11 years old) participated in the two biomonitoring campaigns, carried out in early summer 2017 and winter 2018, respectively. Meteorological conditions were collected during the two days of biomonitoring surveys (summer monitoring day: temperature range = 21–28 °C; relative humidity = 40%; wind speed = 13 km h^−1^. Winter monitoring day: temperature range = 6–10 °C; relative humidity = 73%; wind speed = 17 km h^−1^).

The details about the study project and the full version of the questionnaire used to collect information from participants were reported in our previous studies [41,42,43,44]. Briefly, in the first stage of the project, all students and their parents received information about the aims and plans of the research, the modalities to compile an ad hoc questionnaire and to collect and store urine samples. The day of sampling, each participant collected the last urine sample before going to sleep in a disposable polypropylene bottle (VWR, Milan, Italy) and stored it at 0−+4 °C, and the parents fulfilled the questionnaire, the informed consent, and the personal data processing authorization. The next morning, each participant brought all the material to school, where the research team was waiting for them. Urine samples were transported by means of refrigerated containers to the laboratory, where a 14 mL aliquot of each sample was transferred into a 20 mL glass vial (Agilent, Santa Clara, CA, USA), added with 3 g of dried NaCl (analytical grade, Carlo Erba, Milan, Italy), and sealed with hole aluminum caps with Teflon-lined septa (silicone/PTFE, 20 mm diameter; Agilent, Santa Clara, CA, USA). Each vial was coded and then frozen at −20 °C until the analysis.

The study was conducted according to the guidelines of the Declaration of Helsinki, and approved by the Local Ethics Committee (Azienda Ospedaliero Universitaria Policlinico Umberto I of Rome, protocol code n. 2894).

### 2.2. Covariates and Exposure Information

The questionnaire was specifically designed to investigate on possible factors associated with exposure to traffic derived pollution in childhood. In particular, it provided background information about age, gender, ponderal status, educational levels of parents and the features of indoor environments where the children live (i.e., house density, domestic heating devices). Besides this, daily activities performed during the sampling day (i.e., extra-curricular sport activities, time spent on motor vehicles), the exposure to environmental tobacco smoke (ETS) and information about drinking and food habits (i.e., type of water consumption and the lunch at school) were obtained by the questionnaire.

### 2.3. Urinary Samples Analyses

u-MTBE, u-ETBE, u-TAME, and u-DIPE were determined by headspace solid phase microextraction (SPME) followed by gas chromatography-mass spectrometry (GC-MS) analysis [45]. Briefly, about 1 L of urinary matrix was prepared by pooling urine samples collected from children with low levels of exposure to the monitored compounds, as previously verified. The urine mixture was flushed with nitrogen gas (99.9999% *v/v* purity grade; Alphagaz, Milan, Italy) to further decrease the pollutants concentration and then used for blank tests, calibration, and working standards. A solution of MTBE-d_3_ in methanol (≥99%, Sigma-Aldrich, Milan, Italy) was used as internal standard for the quantification of analytes. Before analysis, each biological sample was thawed out at room temperature, spiked with 2 μL of internal standard solution and then conditioned at 30 °C for 60 min in an aluminum block device (VWR, Milan, Italy). The urine headspace was sampled for 15 min by an 85 µm carboxen/polydimethylsiloxane SPME fiber (Supelco, Bellafonte, PA, USA) and injected into the GC-MS system (Trace GC ULTRA coupled to Focus DSQ MS, Thermo Fisher Scientific, Waltham, MA, USA) operating in SIM mode. Analytes separation was performed on a SPB-624 capillary column (30 m × 0.25 mm × 1.4 μm film thickness; Supelco) using helium (purity grade ≥ 99.9999% *v/v*) as carrier gas at a constant flow rate of 1 ml min^−1^. The split/splitless injection port operated in the splitless mode at 240 °C. The oven temperature program was as follows: 35 °C held for 5 min, ramped to 50 °C at 3 °C min^–1^, then ramped to 230 °C at 30 °C min^–1^ held for 4 min (total run time 20 min). Quantification limits (LOQs), calculated as the arithmetic mean of 15 sample blanks values plus 10 times the standard deviation, were 5.4 ng L^−1^ for MTBE, 5.3 ng L^−1^ for ETBE, 2.9 ng L^−1^ for TAME and 6.1 ng L^−1^ for DIPE. Only the results higher than respective LOQs were considered for statistical analysis.

### 2.4. Statistical Analyses

The investigated variables were coded as follows: gender (0 = male; 1= female); age (0 ≤ 8 years; 1 > 8 years) based on the age of puberty onset [46]; ponderal status according to sex-specific body mass index-for-age growth charts of the United States Centers for Disease Control and Prevention [47] (0 = underweight and normal weight, 1 = overweight and obesity); educational level of each parent according to the Organization for Economic Co-operation and Development [48] (0 = upper secondary or lower, ≤14 years; 1 = tertiary/higher, >14 years); exposure to ETS in domestic environment (0 = no; 1 = yes, one cohabitant smoker at least); house density measured as m^2^ inhabitant^−1^ (0 = high density, ≤30 m^2^ inhab^−1^; 1 = low density, >30 m^2^ inhab^−1^); domestic heating devices (0 = methane or electric heaters, 1 = oil or wood heaters); extra-curricular sport activities (0 = no activity, 1 = at least one activity); time spent on motor vehicles in the sampling day (0 = less than 60 min; 1 = more or equal to 60 min); lunch at school in the sampling day (0 = no; 1 = yes); water consumption habits (0 = only tap water; 1 = only mineral bottled water).

The database was statistically processed using IBM SPSS Statistics software package (version 25, IBM Corp., Armonk, NY, USA). An initial examination of the measured urinary concentrations showed that biomarkers levels were not normally distributed and followed a log-normal distribution according to the Kolmogorov–Smirnov test. Further statistical elaboration was performed applying parametric tests (Student-t for independent samples, and Pearson’s correlation between the urinary excretion profiles of target analytes) on ln-transformed values. *p*-values were based on two-sided tests and significance was set at *p*-value < 0.05. Analytical data were included in a multiple linear regression model to evaluate the influence of some selected variables on the urinary levels of target analytes and their sum as dependent variables. The variables were chosen from those for which it has been verified their statistically significant influence on the biomarkers excretion profile in the univariate analysis.

## 3. Results

### 3.1. Study Population

The summer and winter biomonitoring campaigns achieved a participation rate of about 73% (378 out of 520 recruited healthy children) and 70% (358 out of 510), respectively. From the total amount of participants, few specimens were excluded for an inadequate urine quantity (369 analyzed samples in summer and 294 in winter). In addition, only children of Caucasian origin were included in the study to avoid potential confounder factors (i.e., genetic, metabolic, etc) that may influence exposure biomarker concentrations induced by ethnicity of target population [49], leading to a final number of samples equal to 313 in summer (52.1% female) and 263 in winter (44.9% female). Table 1 summarizes the characteristics of participants in the two seasons.

In both surveys, the mean age was about 9 years (8.68 in summer and 8.89 in winter). In the summer survey, 58.2% children presented a healthy weight (underweight plus normal weight) against the 54.0% rate of winter season. Within the family, the mother had generally a higher level of education than the father (32.9% vs. 23.9% in the summer; 30.4% vs. 22.4% in the winter), while the percentage of ETS exposure was similar in the two studies (about 40% of children were exposed). In both seasons, the largest sample came from the urban site (54.3% in summer and 51.0% in winter). No substantial differences were observed in the residential features between the two seasons: most of participants declared a house density ≤ 30 m^2^ per inhabitant (about 65% in summer and 62% in winter). About 27% of children lived in houses with oil or wood heaters. With regard to the activities carried out on the day of sampling, although a limited number of children practiced at least one extra-curricular sports activity in both seasons, a higher percentage was observed in summer (39.6% vs. 34.6%). In both seasons, most of children were transported by vehicles for a time < 60 min (69.3% in summer and 46.0% in winter). In the summer season, the majority of children ate at home (68.0%) and preferred to drink bottled mineral water (63.9%), while in the winter season 53.6% of participants had their lunch at school and declared to drink more tap-water than in summer (52.9% in the winter vs. 39.0% in the summer). In this season, the percentage of water drunk was similar for the two types (52.9% tap water vs. 56.3% bottled water).

### 3.2. Analytical Determination

Table 2 reports the mean u-concentrations of target gasoline additives found for monitored children grouped according to the variables selected for the data elaboration.

u-DIPE concentrations resulted all under the LOQ values. Besides this, u-TAME mean levels were all above the LOQ, but very similar in the two seasons (18.7 ng L^−1^ in summer vs. 18.9 ng L^−1^ in winter) and in the same season considering the children grouped according to the selected variables. Thus, we reported only data on u-MTBE and u-ETBE and their sum. Statistically significant results, summarized by *p*-values, are evidenced in Table 2.

The most noticeable result is that u-MTBE and u-ETBE concentrations of the entire population were significantly greater in summer (69.9 ng L^−1^ vs. 53.3 ng L^−1^ and 423.5 ng L^−1^ vs. 66.2 ng L^−1^, respectively; *p* < 0.001 for both target analytes). The same feature, even if not statistically significant, was found in all the subgroups.

Another important finding is related to the influence of the time spent in a motor vehicle during the sampling day: those who spent more than 60 min have a significant higher levels of u-MTBE, u-ETBE, and Σ both in summer and winter seasons.

With regard to summer, significant higher levels were found for males (u-ETBE and Σ) and for those who consumed bottled water (u-MTBE, u-ETBE and Σ). In the winter season, we observed significant higher values for younger children (u-ETBE and Σ), overweight and obese (u-ETBE) and those who did not practice any sport during the sampling day (u-ETBE).

Table 3 reports Pearson’s correlation coefficients between u-MTBE and u-ETBE in children grouped according to the sampling season (summer or winter) (a) and the significant predictors of urinary excretion of biomarkers of traffic-related pollutants according to multiple linear regression model with related β and *p* values (b).

Pearson’s correlation analysis performed on entire children population (Table 3, section a) showed a strong positive and statistically significant correlation between the two biomarkers both in summer (r= 0.719, *p* < 0.0001) and in winter (r= 0.165, *p* = 0.011).

Multiple linear regression analysis models partially confirm the results of univariate analysis. In particular, in summer, u-MTBE levels resulted influenced by the time spent in motor vehicles and the kind of water consumption; these variables explain 22.5% of the variability of MTBE (*p* = 0.004). On the contrary, no significant predictors were found for u-MTBE in the winter season. Regarding u-ETBE, in summer their levels were influenced by the time spent in motor vehicles, the kind of water consumption, and the gender, which explains 29.6% of the variability (*p* < 0.001). In the winter season, time spent in motor vehicles and sport activity resulted significant independent predictors of u-ETBE. Finally, the sum of the two biomarkers resulted influenced, in the summer season, by the time spent in motor vehicles, the kind of water consumption, and the gender (29.2% of the variability; *p* < 0.001), and in the winter season by the time spent in motor vehicles and the gender (20.7% of the variability; *p* < 0.023).

## 4. Discussion

The present study was performed to trace an exposure profile to traffic-derived pollution of healthy children by the use of u-MTBE, u-ETBE, u-TAME, and u-DIPE as biomarkers of exposure. u-DIPE resulted always <LOQ demonstrating that this compound is not or little used as a gasoline additive in Italy. u-TAME determinations did not reveal differences between summer and winter or for children grouped according to the selected variable. Both these results are of particular interest, as in our knowledge it is the first biomonitoring study in children for these biomarkers. As regards the other target analytes, children resulted exposed to u-MTBE and u-ETBE at very variable levels. Accordingly, Fustinoni et al. [31] also found variable concentrations of u-MTBE and u-ETBE among not occupationally exposed adults in northern Italy, with median values in the same order of magnitude respect to our results (397 and 74 ng L^−1^, respectively). Another research, performed in Italy on children living near and far from a refinery, reported u-MTBE geometric mean concentration equal to 790 and 560 ng L^−1^, respectively [33]. A study carried out in Iran on adults not occupationally exposed shows u-MTBE, u-ETBE, and u-TAME values equal to 324, 59, and 11 ng L^−1^, respectively [29]. Notice that our data evidences a different exposure profile, where u-ETBE is the biomarker recovered in greatest amount, especially in the summer season. This result can be attributed to the different fuel technical specification that may occur among different countries, even between EU Member States, and for the same country over the years.

Another important evidence resulted from the present study is that u-ETBE and u-MTBE concentrations were significantly higher in the summer than in the winter season. This result agrees with those reported previously, finding higher airborne and u-MTBE concentrations in the warmer season [14,15,18]. The explanation to this finding is difficult because the literature is very scarce. For example, Legreid et al. associated the seasonal variation of oxygenated organic volatile compounds airborne concentrations with lower mean value in summer compared to that in winter with mostly anthropogenic sources [12]. In our case, a plausible hypothesis could be related to the most time spent outside home and/or at home with windows open during summer in Italy.

In both seasons, the concentrations of u-ETBE and u-MTBE and their sum are significantly positively correlated to the time spent in motor vehicles during the sampling day. This result confirms the effectiveness of those compounds as traffic-related pollution biomarkers also during pediatric age.

Another significant predictor for u-ETBE and u-MTBE concentrations and their sum was the consumption of bottled water during the sampling day in summer. Based on that bottled waters do not contain any environmental pollutant according to current European legislation, this result should be associated to other variables linked to the bottled water consumption and needs further investigation.

With regard to gender differences recovered for u-ETBE in summer and for the sum both in summer and winter, other studies reported a significant influence of gender on urinary excretion of several biomarkers during pediatric age [50]. These differences can be attributed to hormonal or genetic factors regulating absorption, distribution, retention, and/or excretion of the biomarkers, but this hypothesis warrants future confirmation.

Unexpectedly, the place of residence did not affect the exposure profile; probably, the differences between urban and rural areas in terms of airborne pollution in the studied area are not so relevant to significantly influence the urinary excretion of the target analytes.

The present study had some limitations. Firstly, we did not perform measurements of airborne levels of the studied compounds in the living environments of the participants and, consequently, a correlation between air concentration and intake could not be defined; thus, the interpretation of our findings should be careful and conservative. 

Secondly, only one urine sample was collected for each participant (just before bedtime); thus, possible changes in biomarkers excretion within that time could not be evaluated. Finally, the multivariate model explains only a part of the variance in u-MTBE and u-ETBE levels and their sum, suggesting that other variables (e.g. genetic polymorphisms, food intake, etc) can influence the urinary excretion of the monitored compounds; these additional factors should be investigated in future research in this field.

## 5. Conclusions

In our biomonitoring surveys, u-MTBE and u-ETBE levels of a large sample of children resulted closely related both to each other and to time spent on motor vehicles, demonstrating their use as reliable biomarkers of traffic exposure during pediatric age. 

Other important findings that need for further investigation are the influence of the summer season itself or related to peculiar summer habits, and the consumption of bottled water. Besides this, it should be important to perform the same biomonitoring study on children living in areas with stronger differences in airborne pollution, to further investigate it.

On a public health point of view, our results highlight that children are exposed at measurable levels of traffic-derived airborne pollution that is a well-known risk factor for human health. This is of particular importance because children are a highly susceptible population to the adverse effects of environmental contaminants. In order to increase the scientific evidence on this issue, human biomonitoring studies tracing exposure profiles to air pollution during pediatric age are needed in many countries.

## Figures and Tables

**Table 1:** Characteristics of the study population participating to the summer and winter biomonitoring campaigns (2017–2018).

		Descriptive in% If Not Stated Otherwise (*n*)			
Variable		Summer 2017	*p*-Value ^1^	Winter 2018	*p*-Value ^1^
		(*n* = 313)		(*n* = 263)	
Age ^2^		Mean 8.68 (standard deviation 1.48)	-	Mean 8.89 (standard	-
				deviation 4.32)	
Gender ^2^	Male	47.9 (*n* = 150)	0.498	55.1 (*n* = 145)	0.033
	Female	52.1 (*n* =163)		44.9 (*n* = 118)	
Ponderal status according to BMI ^3^	Underweight	7.7 (*n* = 24)	<0.001	7.2 (*n* = 19)	<0.001
	Normal weight	50.5 (*n* = 158)		46.8 (*n* = 123)	
	Overweight	16.9 (*n* = 53)		15.2 (*n* = 40)	
	Obesity	11.5 (*n* = 36)		14.5 (*n* = 38)	
	Unknown	13.4 (*n* = 42)		16.3 (*n* = 43)	
Maternal education (years)	Basic (≤9 years)	16.0 (*n* = 50)	<0.001	16.4 (*n* = 43)	<0.001
	Upper secondary (≤14 years)	47.6 (*n* = 149)		49.8 (*n* = 131)	
	Tertiary/higher (>14 years)	32.9 (*n* = 103)		30.4 (*n* = 80)	
	Unknown	3.5 (*n* = 11)		3.4 (*n* = 9)	
Paternal education (years)	Basic (≤9 years)	25.9 (*n* = 81)	<0.001	24.0 (*n* = 63)	<0.001
	Upper secondary (≤14 years)	45.4 (*n* = 142)		50.6 (*n* = 133)	
	Tertiary/higher (>14 years)	23.9 (*n* = 75)		22.4 (*n* = 59)	
	Unknown	4.8 (*n* = 15)		3.0 (*n* = 8)	
Exposure to ETS ^4^	Not exposed	58.5 (*n* = 183)	0.002	59.3 (*n* = 156)	<0.001
	Exposed	40.6 (*n* =127)		38.0 (*n* = 100)	
	Unknown	0.9 (*n* = 3)		2.7 (*n* = 7)	
Place of residence	Rural areas	42.2 (*n* = 132)	0.096	47.9 (*n* = 126)	0.123
	Urban area	54.3 (*n* = 170)		51.0 (*n* = 134)	
	Unknown	3.5 (*n* = 11)		1.1 (*n* = 3)	
House density (m^2^ inhabitant^−1^)	≤30	65.2 (*n* = 204)	<0.001	61.6 (*n* = 162)	<0.001
	>30	30.0 (*n* = 94)		33.5 (*n* = 88)	
	Unknown	4.8 (*n* = 15)		4.9 (*n* = 13)	
Domestic heating devices	Methane or electric heaters		-	68.8 (*n* = 181)	0.287
	Oil or wood heaters			27.4 (*n* = 72)	
	Unknown			3.8 (*n* = 10)	
Extra-curricular sport activities ^5^	No	59.1 (*n* = 185)	<0.001	63.1 (*n* = 166)	<0.001
	Yes	39.6 (*n* = 124)		34.6 (*n* = 91)	
	Unknown	1.3 (*n* = 4)		2.3 (*n* = 6)	
Time spent on vehicles ^5^	0 min	28.4 (*n* = 89)	<0.001	0.0 (*n* = 0)	<0.001
	<60 min	40.9 (*n* = 128)		46.0 (*n* = 121)	
	≥60 min	27.5 (*n* = 86)		29.3 (*n* = 77)	
	Unknown	3.2 (*n* = 10)		24.7 (*n* = 65)	
School lunch ^5^	No	68.0 (*n* = 213)	<0.001	43.0 (*n* = 113)	0.079
	Yes	30.7 (*n* = 96)		53.6 (*n* = 141)	
	Unknown	1.3 (*n* = 4)		3.4 (*n* = 9)	
Tap water ^5,6^	No	59.4 (*n* = 186)	<0.001	44.1 (*n* = 116)	0.002
	Yes	39.0 (*n* = 122)		52.9 (*n* = 139)	
	Unknown	1.6 (*n* = 5)		3.0 (*n* = 8)	
Bottled water ^5^	No	34.5 (*n* = 108)	0.386	40.3 (*n* = 106)	0.019
	Yes	63.9 (*n* = 200)		56.3 (*n* = 148)	
	Unknown	1.6 (*n* = 5)		3.4 (*n* = 9)	

^1^ Chi square test. ^2^ Unknown = 0. ^3^ BMI: body mass index (weight in kg/height squared in m). ^4^ ETS: environmental tobacco smoke. ^5^ During the sampling day. ^6^ Including drinking water treated at home (e.g., added with CO_2_).

**Table 2:** Urinary concentrations (ng L^−1^) of methyl tert-butyl ether (MTBE), ethyl tert-butyl ether (ETBE), and their sum (Σ), in children grouped according to some personal characteristics, their living environments and activity carried out in the sampling day. Values are expressed as arithmetic mean (AM) and standard deviation (SD).

		Summer 2017			Winter 2018		
		u-MTBE AM (SD)	u-ETBE AM (SD)	Σ	u-MTBE AM (SD)	u-ETBE AM (SD)	Σ
Entire population		69.9 (56.4)	423.5 (343.5) **	493.4 (377.7)	53.3 (46.8)	66.2 (61.7) **	119.5 (83.6)
Gender	Male	70.2 (46.5)	466.0 (358.6) *	536.2 (391.2) *	55.7 (46.8)	62.9 (56.2)	118.6 (75.5)
	Female	69.5 (64.6)	382.4 (324.3)	451.9 (360.8)	50.4 (47.3)	70.0 (67.7)	120.4 (93.2)
Age	≤8	70.1 (60.7)	446.3 (370.3)	516.7 (408.6)	57.0 (50.6)	74.9 (71.2) *	131.9 (96.8) *
	9–11	69.6 (51.0)	404.2 (319.0)	473.8 (349.6)	49.9 (42.9)	58.5 (50.0)	108.4 (67.4)
Ponderal status according to BMI	Under/healthy weight	69.9 (56.4)	423.4 (282.6)	493.3 (311.5)	51.9 (44.4)	61.6 (61.8) *	113.5 (79.1)
	Overweight or obese	70.3 (46.0)	445.4 (386.8)	515.7 (423.1)	57.0 (50.5)	74.0 (62.4)	131.0 (92.1)
Maternal education (years)	Upper secondary or lower	68.1 (52.3)	422.4 (336.7)	490.5 (370.5)	52.9 (48.0)	64.4 (59.9)	117.3 (80.1)
	Tertiary/higher	73.4 (64.1)	425.6 (358.7)	499.0 (393.8)	54.0 (44.0)	70.4 (66.1)	124.4 (91.9)
Paternal education (years)	Upper secondary or lower	70.2 (52.8)	442.0 (378.0)	512.2 (414.2)	52.9 (46.1)	66.4 (58.8)	119.3 (78.5)
	Tertiary/higher	68.9 (66.3)	368.1 (199.7)	437.0 (229.1)	54.2 (49.0)	65.3 (70.5)	119.5 (98.8)
Exposure to ETS	Not exposed	73.9 (64.0)	416.6 (328.8)	490.5 (355.5)	54.3 (44.5)	66.7 (58.0)	121.0 (65.2)
	Exposed	63.9 (42.2)	433.6 (365.8)	497.5 (395.8)	51.6 (41.3)	65.1 (52.8)	116.7 (60.5)
Place of residence	Rural area	61.5 (42.2) *	403.7 (311.3)	465.2 (320.9)	48.6 (43.0)	70.0 (57.0)	118.6 (65.1)
	Urban site	78.2 (66.4)	445.2 (376.8)	523.4 (395.8)	57.8 (50.9)	62.4 (52.6)	120.2 (63.4)
House density	≤30	73.4 (59.7)	435.5 (364.0)	508.9 (399.6)	50.3 (42.5)	59.7 (54.3)	110.0 (71.4)
	>30	64.4 (50.4)	398.9 (308.1)	463.3 (340.5)	59.6 (53.1)	77.0 (70.1)	136.6 (93.6)
Extra-curricular sport activities	No	67.8 (52.6)	403.5 (306.9)	471.3 (340.6)	54.8 (48.6)	70.4 (66.9) *	125.2 (89.8)
	Yes	72.8 (62.5)	455.1 (395.4)	527.9 (430.9)	50.3 (43.6)	58.9 (49.7)	109.2 (69.7)
Time spent on vehicles	<60 min	66.1 (55.8) *	397.6 (317.1) **	463.7 (349.7) **	54.7 (50.6) *	55.6 (48.9) *	110.3 (75.1) *
	≥60 min	80.7 (48.7)	532.2 (422.9)	612.9 (462.2)	61.3 (46.1)	79.4 (73.1)	140.7 (99.7)
School lunch	No	73.9 (63.9)	439.5 (376.9)	513.4 (415.1)	59.2 (49.4)	68.1 (68.2)	127.3 (93.2)
	Yes	60.5 (34.1)	390.2 (263.0)	450.7 (285.4)	47.3 (42.9)	64.8 (57.2)	112.1 (75.6)
Kind of water consumption	Only tap water	62.6 (53.4) *	370.3 (251.7) *	432.9 (280.2) *	54.3 (48.2)	74.8 (71.1)	128.6 (97.2)
	Only bottled mineral water	78.3 (54.7)	489.8 (430.6)	568.1 (467.0)	49.5 (43.3)	65.7 (59.3)	115.2 (74.4)
Domestic heating device	Methane or electric heaters	-	-	-	55.1 (47.7)	61.9 (49.6)	117.0 (74.5)
	Oil or wood heaters	-	-	-	52.3 (46.8)	71.8 (71.2)	124.1 (92.1)

* *p* ≤ 0.05. ** *p* ≤ 0.001.

**Table 3:** (a) Pearson’s correlation coefficients between u-MTBE and u-ETBE in children classified by sampling season (summer and winter); (b) Significant predictors of urinary excretion of biomarkers of traffic-related pollutants according to multiple linear regression model.

			Summer		Winter	
				u-MTBE		
a		u-ETBE	0.719		0.165	
			(*p* < 0.0001)		(*p* = 0.011)	
			**Summer**		**Winter ^1^**	
	Model	u-MTBE	u-ETBE	u-Σ	u-ETBE	u-Σ
		R = 0.225	R = 0.296	R = 0.292	R = 0.247	R = 0.207
		*p* = 0.004	*p* < 0.0001	*p* < 0.0001	*p* = 0.005	*p* = 0.023
	Constant	3.857	5.749	5.908	3.889	4.584
	Time in motor vehicles	0.228	0.327	0.308	0.223	0.193
		−0.031	−0.002	−0.002	−0.05	−0.05
b	Type of water consumed during the sampling day	0.224	0.203	0.208		
		−0.013	−0.023	−0.016		
	Gender		−0.189 (0.030)	−0.167 (0.047)		−0.19
						−0.048
	Sport activity carried out during the sampling day				−0.289 (0.008)	

^1^ no significant predictors were found in the winter season for u-MTBE.

## Data Availability

Data have been provided as tables and figures directly within the manuscript, and raw data are available via e-mail upon request to the corresponding author.
